# A Novel Homozygous *ADCY5* Variant is Associated with a Neurodevelopmental Disorder and Movement Abnormalities

**DOI:** 10.1002/mdc3.13310

**Published:** 2021-07-31

**Authors:** Rauan Kaiyrzhanov, Maha S. Zaki, Reza Maroofian, Natalia Dominik, Aboulfazl Rad, Barbara Vona, Henry Houlden

**Affiliations:** ^1^ Department of Neuromuscular Disorders University College London, Institute of Neurology London United Kingdom; ^2^ Human Genetics and Genome Research Division, Clinical Genetics Department National Research Centre Cairo Egypt; ^3^ Department of Otolaryngology—Head & Neck Surgery Tübingen Hearing Research Centre, Eberhard Karls University Tübingen Tübingen Germany

**Keywords:** dystonia, autosomal recessive, *ADCY5*, neurodevelopmental disorder, movement disorders

The genetic and clinical spectrum of adenylyl cyclase 5 (*ADCY5*)‐related disease has considerably been expanding in recent years. To date, from over 70 *ADCY5*‐related disease reports, only three have been associated with an autosomal recessive (AR) inheritance, while the rest of the reports were linked to an autosomal dominant (AD) inheritance.^1^, ^2^, ^3^, ^4^ Clinical comparison of the cases with different modes of inheritance in *ADCY5‐*related disease is currently challenging due to the paucity of reports on the AR form and broad phenotype of the AD form. Herein, we report a family with an AR *ADCY5‐*related disease.

Three affected siblings, including two females and one male, were born to the non‐consanguineous parents originating from the same Egyptian village (Fig. 1A). The elder affected sibling (III‐2) died at the age of 4 years old, and the younger affected siblings are currently aged 8 (III‐5) and 2 (III‐6) years old. All were the products of an uneventful full‐term pregnancy and delivery with normal postnatal measurements. The disease started with global developmental delay, axial hypotonia, and appendicular spasticity. By the ages of 7 and 9 months, all affected siblings developed intermittent limb dyskinesia and dystonia (Tables S1 and S2 for detailed clinical information). Slightly later, they had started displaying abnormal eye movements and facial twitches. Limb spasticity and dystonia had a progressive course with dystonia gradually becoming constant and severe, as could be seen in the older sibling (III‐5) aged 8 years old (Fig. 1D and Video 1 for III‐5 and III‐6). The hyperkinetic movements persisted with no alleviation from sleep and tended to exacerbate in a paroxysmal manner. Occasionally, there had been motor exacerbations in arousal. Episodes of obsessive–compulsive behavior with anxiety, phobias, and grinding movements were commonly displayed behavioral signs. Two siblings (III‐2 and III‐5) developed bouts of laughing and crying leading to apneic spells.

**FIG. 1:**
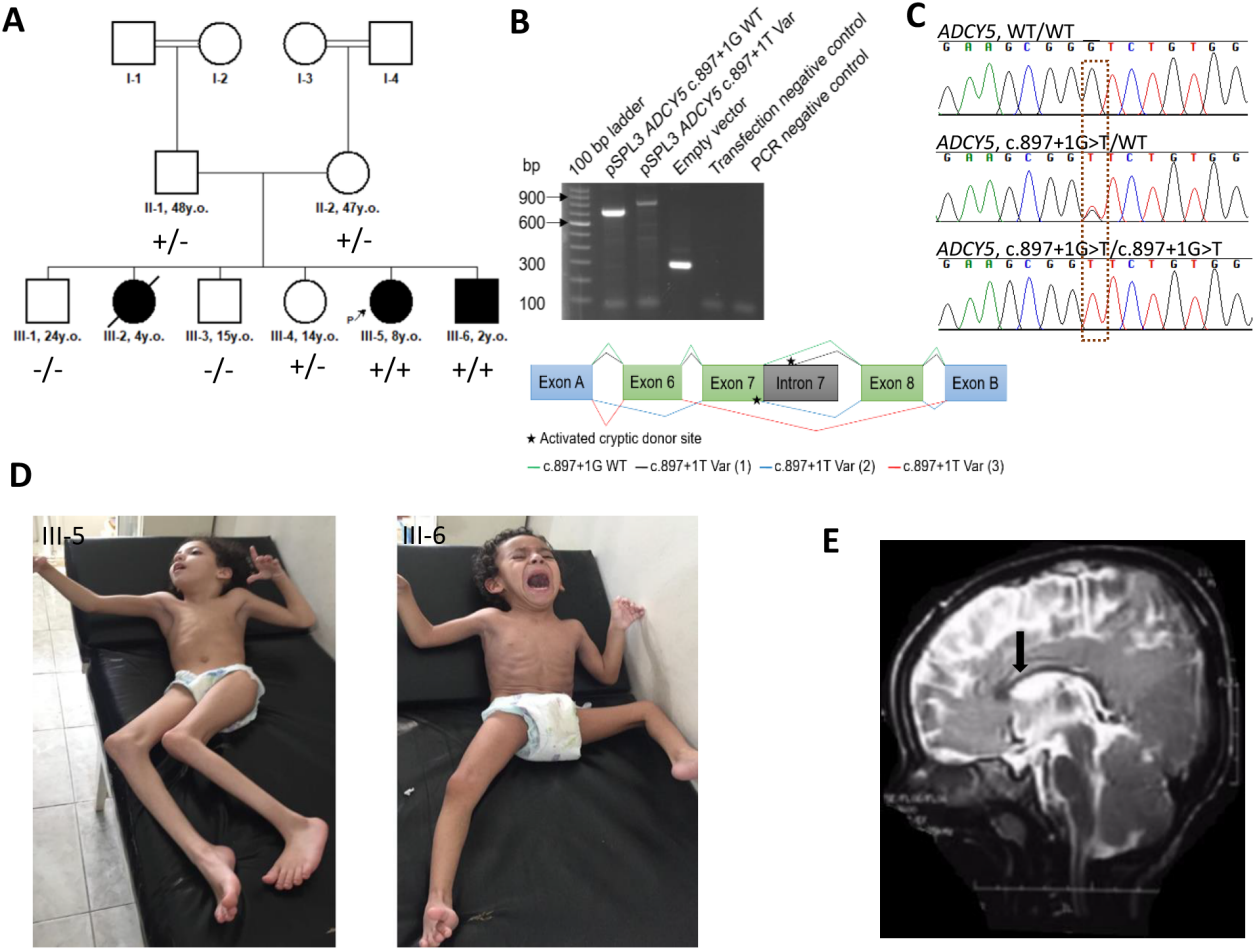
Pedigree, genetics, and clinical features of the presented individuals. (A) Family tree with the genotypes of the proband, affected sibling, unaffected siblings, and parents. Square‐ male; circle‐ female, black symbols‐ affected individuals, white symbols‐ unaffected carriers. “+” – *ADCY5* c.897+1G>T variant, “‐”‐ wild type. (B) Electrophoretic visualization of minigene RT‐PCR products (left). Schematic illustration of the minigene splice construct design and splicing results consisting of exon trapping vector exons A and B (blue), exons 6–8 (green) and partial intron 7 (grey) (right). Stars represent activated cryptic donor sites. Splicing schematic of the WT (green) and three aberrantly spliced amplicons (Var1‐3, grey, blue, orange, respectively) are shown with lines. Abbreviations: Var, variant; WT, wild‐type. (C) Sanger chromatograms of the family members with the homozygous (III‐5 and III‐6) and heterozygous (II‐1, II‐2, and III‐4) *ADCY5* c.897+1G>T substitution along with the carriers of wild type alleles (III‐1 and III‐3). (D) Images of the affected siblings (III‐5 and III‐6). (E) Sagittal brain MRI image of the youngest affected sibling (III‐6) showing mild corpus callosum hypoplasia.

**Video 1:** Video is available through the following link: https://www.dropbox.com/s/k1y5i2pj857k993/Supplementary%20video.mp4?dl=0/. *Segment 1* shows the proband (III‐5) with generalized dystonia, spasticity, and knee/elbow joint contractures. *Segment 2* shows the proband sitting leaning towards the wall. She has dystonia in her fingers and the left foot. *Segment 3* shows dystonia, increased muscle tone, and severe contractures at the elbows and knees in the proband. *Segment 4* Shows increased tendon reflexes from the upper limbs in the proband. She does not follow the hummer with her eyes when checking pursuit. *Segment 5* shows that proband cannot stand and walk. *Segment 6* shows upward tonic eye deviation in the affected sibling, very subtle twitching of the perioral muscles, and one myoclonic head jerk (III‐6). *Segment 7* shows the inability to sit independently, axial hypotonia, and no head control in the affected sibling. Periodic myoclonic jerks are also visible in the upper limbs (left more than right) together with dyskinetic movements in all limbs. *Segment 8* shows limb dystonia, increased muscle tone, and brisk tendon reflexes in the affected sibling.

Upon the last follow‐up examinations at the ages of 4, 8, and 2 years, the siblings had globally unachieved milestones with head circumferences, weights, and heights below the 5th percentile. They were non‐verbal with severe intellectual disability and were not able to understand simple instructions. Limb dystonia and spasticity leading to joint contractures were more prominent in the older siblings. The youngest sibling (III‐6) had subtle twitching of the perioral muscles, dyskinetic limb movements, and mild superimposed myoclonic jerks in the upper limbs. He also displayed intermittent upward tonic eye deviations. Tremor, persisting axial hypotonia, and brisk tendon reflexes were present in all affected siblings. (Fig. 1D, Video 1 III‐5, III‐6). Cardiomyopathy was found only in the elder sister (III‐2) who succumbed to recurrent chest infections at 4 years old.

A trial of clonazepam, levodopa, biperiden, amantadine, and cyclobenzaprine failed to adequately control hyperkinetic movements and spasticity in all three siblings. The results of comprehensive metabolic panel testing including alpha‐fetoprotein (III‐5, III‐6), lactic acid (all siblings), ceruloplasmin (III‐2, III‐5), 24‐hour urinary copper (III‐2, III‐5) were unremarkable and the possibility of Wilson's disease was ruled out. The eye fundi appeared normal in all affected cases and brain magnetic resonance imaging (MRI) was suggestive of the thin corpus callosum in sibling III‐6 (Fig. 1E). The parents of the affected cases and their unaffected siblings have remained asymptomatic upon the most recent follow‐up.

To identify the genetic cause of the disease in the affected children, exome sequencing (ES) on DNA extracted from proband's blood (III‐5) was performed as previously described.^5^ In accordance with the recessive mode of inheritance, priority was given to rare biallelic functional variants with allele frequency <0.001% in public databases, including 1000 Genomes project, NHLBI Exome Variant Server, Complete Genomics 69, Iranome, GME Variome, and gnomAD as well as our in‐house database consisting of over 20,000 exomes. A novel homozygous splice donor variant in *ADCY5* c.897+1G>T (NM_001199642.1) residing within a 16.8 Mb region of homozygosity was identified, which segregated with the phenotype in the family (Fig. 1A,C, Figs S1 and S2). The variant is predicted to affect splicing between exons 7 and 8 of the *ADCY5* gene that impairs the function of the first catalytic C1 domain the protein (Fig. S3). Minigene testing and TA cloning of the c.897+1T variant identified three aberrantly spliced amplicons: cryptic donor splice site activation of intron 7, extending the amplicon by 171 bp, activation of a cryptic donor splice site in exon 7 and skipping of exon 6, and skipping of exons 7 and 8 (Fig. 1B, Fig. S4). The c.897+1G>T variant in *ADCY5* is classified as “pathogenic” according to The American College of Medical Genetics and Genomics (ACMG) criteria (PVS1, PM2, PP3). No additional rare biallelic variants of likely pathogenic significance were identified (Table S3).

Individuals with *ADCY5‐*related dyskinesia identified to date have overlapping but not identical clinical manifestations with wide‐ranging clinical severity.^6^ Here we report 3 siblings with a homozygous loss‐of‐function (LOF) *ADCY5* variant presenting with the severe end of the *ADCY5*‐related dyskinesia phenotypic spectrum. The cases had distinctive *ADCY5*‐related dyskinesia features including dyskinesia, perioral twitching, ballistic bouts, and motor exacerbations in arousal.^1^, ^6^ Additionally, they had common *ADCY5*‐related dyskinesia signs such as axial hypotonia, spasticity, dystonia, and limb tremor, together with infrequent features including intellectual disability, psychiatric symptoms, and cardiomyopathy. Persisting severe axial hypotonia along with progressive spasticity and dystonia with hyperreflexia seen in the present cases have also been reported in *ADCY5‐*related disease with AD inheritance.^1^, ^6^, ^7^ The typical choreiform movements often characterized as piano playing movements were absent in the present cases. This could be explained by the phenotypic variability of *ADCY5‐*related disease. Of note, *ADCY5*‐related dyskinesia appears to result from either a gain or loss‐of‐function mechanism, although the underlying mechanisms accounting for these differences are not yet fully understood.^7^ The homozygous LOF variant could potentially account for the phenotype severity in the present family. This LOF variant does not seem to express itself into a disease phenotype in the heterozygous state, as both parents and one heterozygous carrier sibling remain disease‐free. This could be explained by the residual enzyme activity conferred by the wild‐type allele.

Two out of three previously reported families with biallelic *ADCY5* variants presented with a rare myoclonus‐dystonia phenotype of the *ADCY5‐*dyskinesia spectrum.^2^, ^3^ The third family with two affected children had severe global developmental delay and dystonia. Mild brain stem hypoplasia was present in one of these siblings.^4^ (Tables S1 and S2). All cases with the myoclonus‐dystonia phenotype, with the age range of 6–27 years old (eight affected individuals), had attained independent ambulation before the age of 2 years despite the reported developmental delay. Their cognitive performance and brain MRIs were reported to be normal and only one of them developed mild joint contractures. Resembling our report, none of the parents of the affected individuals from these three families exhibited movement disorders. Brain MRI shows no evidence of structural abnormalities in *ADCY5* dyskinesia,^6^ however, similar to the report by Okamoto et al.,^4^ we found mild structural brain abnormality including corpus callosum hypoplasia in one of the siblings. These MRI findings would need to be validated in further families with an AR form of *ADCY5‐*related dyskinesia.

A range of neuropsychiatric features associated with *ADCY5* dyskinesia included psychosis, depression, obsessive–compulsive behavior, and anxiety.^8^ Psychosis and depression developed in adult cases, while younger patients had obsessive–compulsive behavior and anxiety, similar to our cases. The presence of neuropsychiatric features in the present family could be explained by the loss‐of‐function nature of the *ADCY5* variant in our study. *ADCY5* knock‐out mice study demonstrated that the loss of ADCY5 in mice produces impairments in sociability and stereotyped behavior.^1^ In contrast to this, *ADCY5* overexpression but not disruption in mice results in a cardiomyopathy.^1^

Further studies are warranted to improve our understanding of the clinical spectrum and genotype–phenotype correlation of *ADCY5*‐related disease.

## Author Roles

(1) Research Project: A. Conception, B. Organization, C. Execution; (2) Manuscript Preparation: A. Writing of the first draft, B. Review and Critique.

R.K.: 1A, 2A, 2B

M.S.Z.: 1B, 2C, 2B

R.M.: 1A, 1B, 2B

N.D.: 1B, 2B

A.R.: 1C, 2B

B.V.: 1C, 2B

H.H.: 1A, 1B, 2B

## Disclosures

### Ethical Compliance Statement

Written informed consent for genetic testing and photo/video materials were obtained from the parents. The study was conducted in accordance with the Declaration of Helsinki and approved by the relevant institutional review boards. We confirm that we have read the Journal's position on issues involved in ethical publication and affirm that this work is consistent with those guidelines.

### Funding Sources and Conflicts of Interest

This study was funded by the Medical research council (MRC) (MR/S01165X/1, MR/S005021/1, G0601943), Intramural Funding (fortüne) at the University of Tübingen (2545‐1‐0 to B.V.), and the Ministry of Science, Research and Art Baden‐Württemberg (to B.V.). The authors declare that there are no conflicts of interest relevant to this work. This research was funded in part, by the Wellcome Trust [Grant number WT093205MA, WT104033AIA and the Synaptopathies Strategic Award, 165,908]. For the purpose of open access, the author has applied a CC BY public copyright licence to any Author Accepted Manuscript version arising from this submission.

### Financial Disclosures for the Previous 12 Months

The authors declare that there are no additional disclosures to report.

## Supporting information

**Figure S1**. Results of autozygosity mapping (A, B). The vcf file from exome sequencing of the proband was analyzed using www.homozygositymapper.org. Figure 1A shows multiple red columns of various widths spread across different chromosomes. These red columns are reflective of genomic regions without heterozygosity, ie all the genetic variations within the regions have two identical alleles. Hence, these regions are called regions of homozygosity (ROH). Multiple large ROH spread across different chromosomes is representative of parental consanguinity. Pathogenic homozygous variants in recessive genes in a proband from a consanguineous family must reside in large ROH (> 5 Mb). Figure 1B shows a table with ROH sizes as several base pairs in each chromosome. The *ADCY5* c.897+1G>T variant has a genomic location 123,327,617 on chromosome 3, which is in the interval between the genomic positions 109,093,307 and 125,922,316 shown in the first raw of the table. To calculate the ROH size, we deduct 109,093,307 from 125,922,316 and this gives us 16,829,009 base pairs, which equals 16.8 Mb.

**Figure S2**. Sanger sequencing electropherograms. First raw—proband (III‐5); second raw—unaffected sister (III‐4); third raw—father (II‐1); fourth raw—mother (II‐2); fifth raw—affected sibling (III‐6); sixth raw—unaffected sibling (III‐1); seventh raw‐unaffected sibling (III‐3).

**Figure S3**. The impact on splicing signals analysis for *ADCY5* c.897+1G>T (A, B). Signal interpretation: Broken WT Donor site alteration of the WT Donor site, most probably affecting splicing. The analysis was performed using https://hsf.genomnis.com/. Introns are non‐protein‐coding DNA sequences and those introns immediately adjacent to exons are important for correct splicing between exons. Splicing regions of introns can be divided into Donor and Acceptor sites. The red arrows on Fig. 1B show the Donor site and the blue arrow shows the Acceptor site between the exons 7–8 of the ADCY5 gene. *ADCY5* c.897+1G>T variant breaks the Donor site.

**Figure S4**. Detailed view of the minigene results. (A) Electrophoretic visualization of RT‐PCR products. Wild‐type splicing (699 bp) consists of Exon A, Exons 6–8, and Exon B. The mutant amplicon shows three faint bands. The empty vector (257 bp), transfection negative and PCR negative controls performed as expected. (B) An overview of the WT, vector control, and aberrantly spliced amplicons. (C) Electropherograms of the RT‐PCR products for wild‐type and aberrant splicing (D) that includes activation of a cryptic splice site in intron 7 (Var 1, 870 bp), skipping of exon 6, and cryptic donor site activation in exon 7 (Var 2, 539 bp), and skipping of exons 7 and 8 (Var 3, 416 bp). (E) Electropherograms of the empty vector control. Abbreviations: Var, variant; WT, wild‐type.

**Appendix S1**. Minigene assay methods.

**Table S1**. Clinical features of the cases with biallelic *ADCY5* variants

**Table S2**. More detailed clinical information for the presented and previously reported families with biallelic *ADCY5* variants. *Available as a separate excel file*.

**Table S3**. Rare homozygous and compound heterozygous variants found in the proband from exome sequencing. *Available as a separate excel file*.
