# Supplementary figures and images for: A Novel Homozygous ADCY5 Variant is Associated with a Neurodevelopmental Disorder and Movement Abnormalities
Source: Mov Disord Clin Pract. 2021 Jul 31;8(7):1140–3. doi: 10.1002/mdc3.13310 (PMC8485619; doi:10.1002/mdc3.13310)

**A**

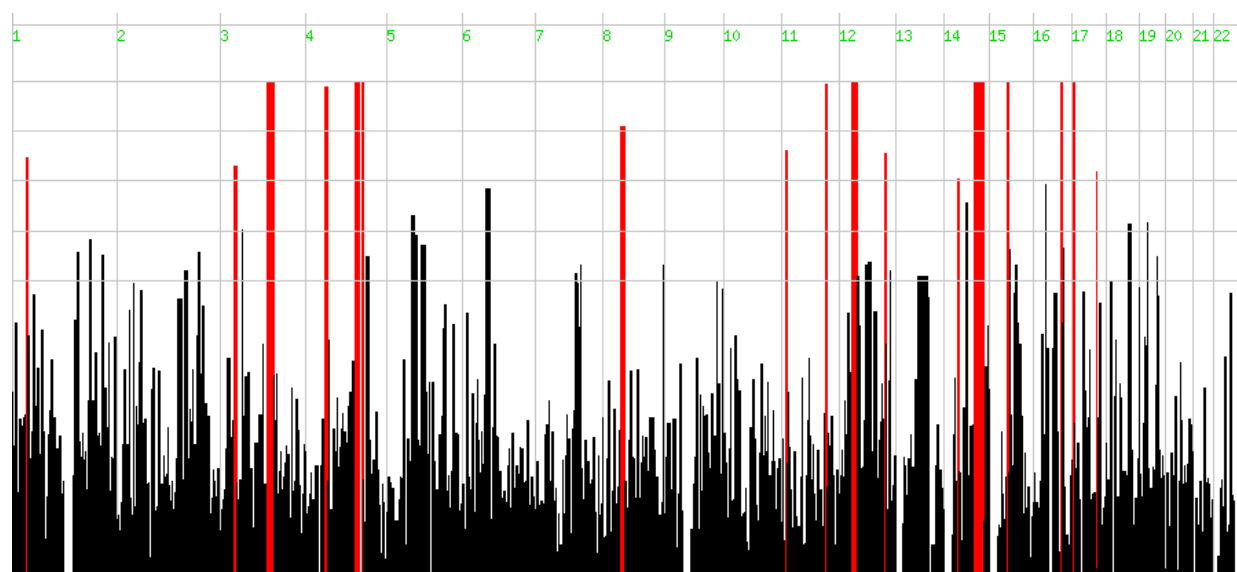

# B

[illegible]

Supplement: Supplementary file 1 — Figure S1. Results of autozygosity mapping (A, B). The vcf file from exome sequencing of the proband was analyzed using www.homozygositymapper.org. Figure 1A shows multiple red columns of various widths spread across different chromosomes. These red columns are reflective of genomic regions without heterozygosity, ie all the genetic variations within the regions have two identical alleles. Hence, these regions are called regions of homozygosity (ROH). Multiple large ROH spread across different chromosomes is representative of parental consanguinity. Pathogenic homozygous variants in recessive genes in a proband from a consanguineous family must reside in large ROH (> 5 Mb). Figure 1B shows a table with ROH sizes as several base pairs in each chromosome. The ADCY5 c.897+1G>T variant has a genomic location 123,327,617 on chromosome 3, which is in the interval between the genomic positions 109,093,307 and 125,922,316 shown in the first raw of the table. To calculate the ROH size, we deduct 109,093,307 from 125,922,316 and this gives us 16,829,009 base pairs, which equals 16.8 Mb. [file MDC3-8-1140-s005.pdf]

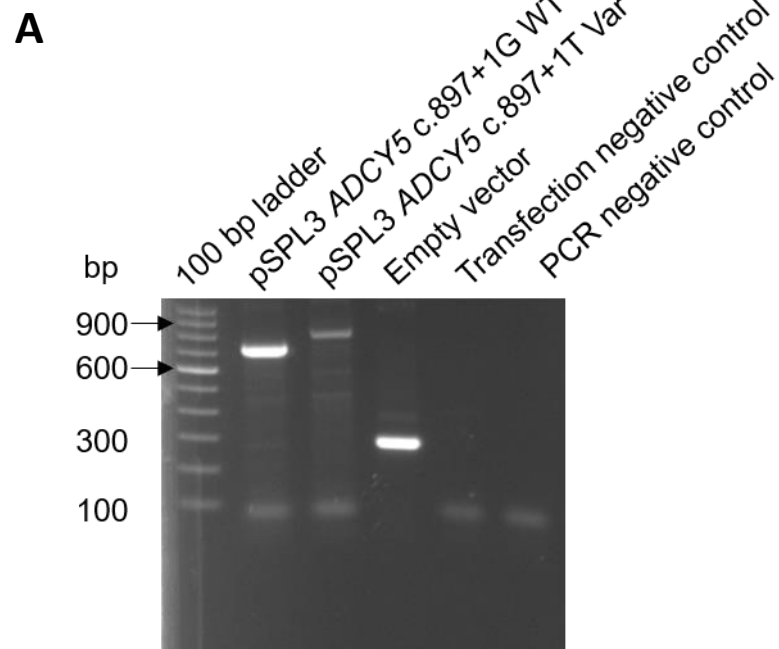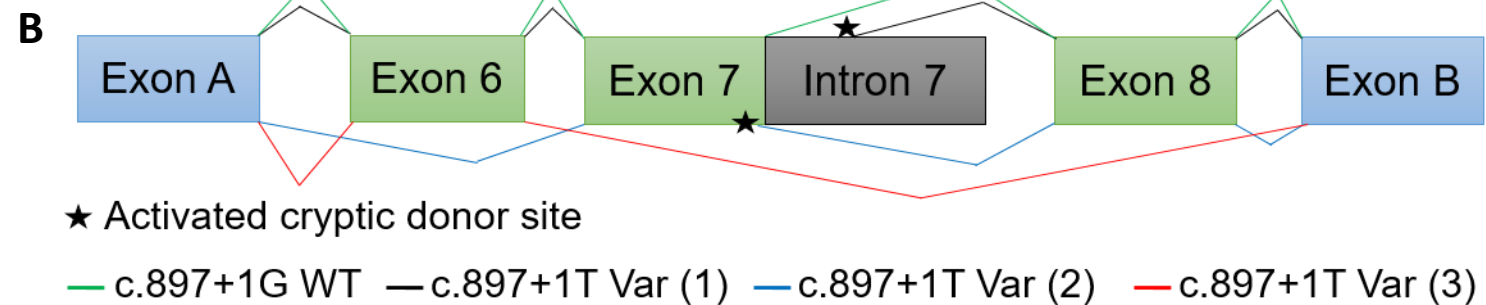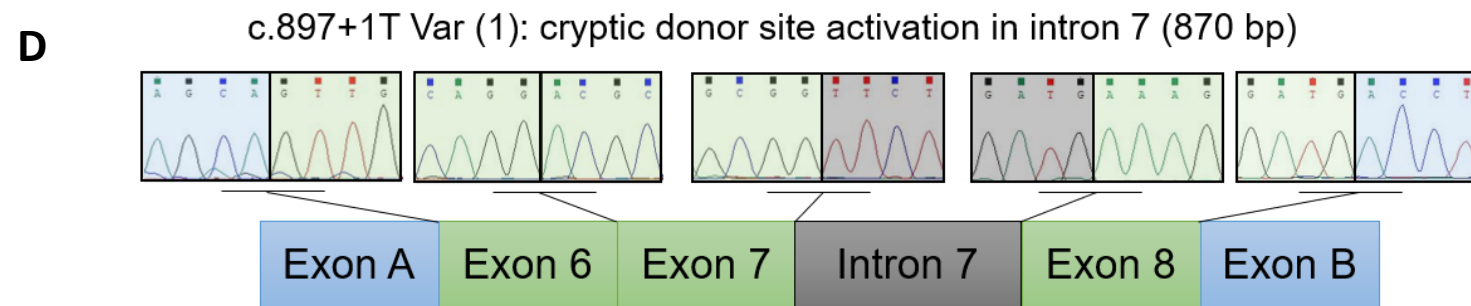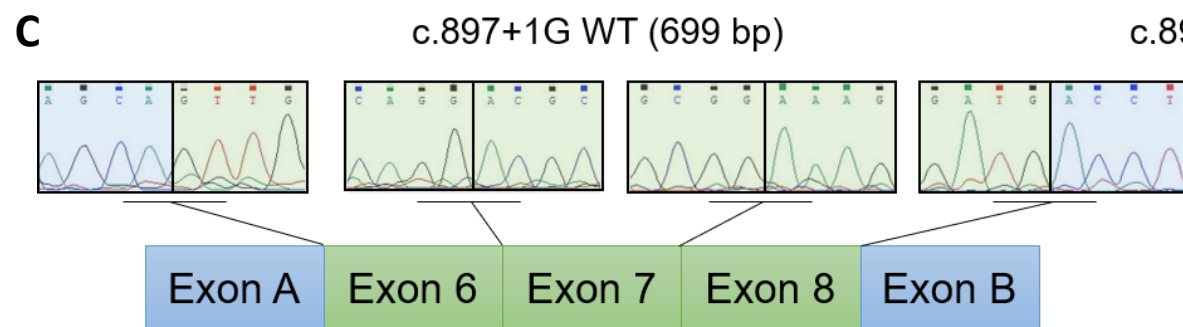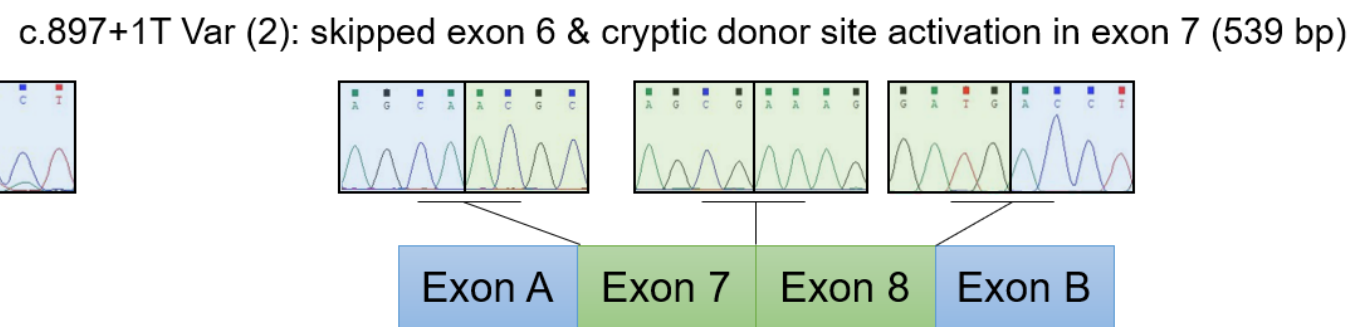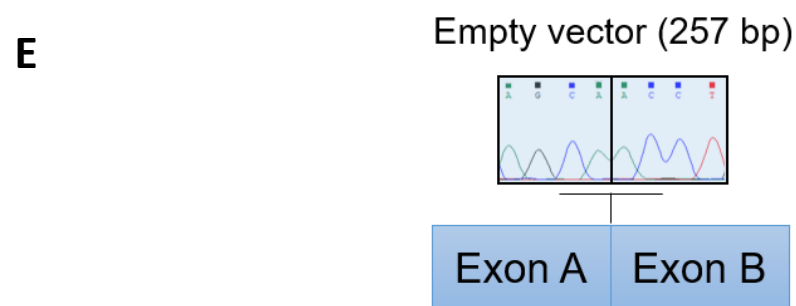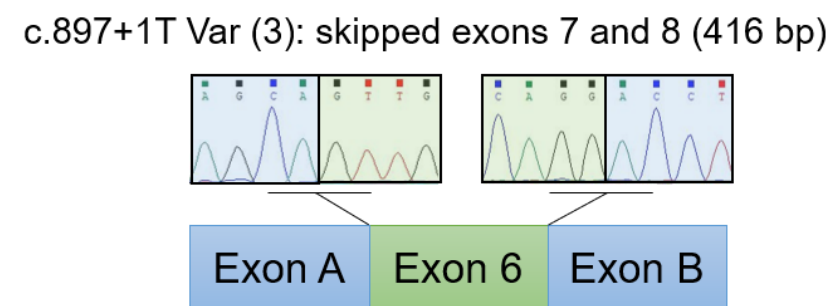

Supplement: Supplementary file 4 — Figure S4. Detailed view of the minigene results. (A) Electrophoretic visualization of RT‐PCR products. Wild‐type splicing (699 bp) consists of Exon A, Exons 6–8, and Exon B. The mutant amplicon shows three faint bands. The empty vector (257 bp), transfection negative and PCR negative controls performed as expected. (B) An overview of the WT, vector control, and aberrantly spliced amplicons. (C) Electropherograms of the RT‐PCR products for wild‐type and aberrant splicing (D) that includes activation of a cryptic splice site in intron 7 (Var 1, 870 bp), skipping of exon 6, and cryptic donor site activation in exon 7 (Var 2, 539 bp), and skipping of exons 7 and 8 (Var 3, 416 bp). (E) Electropherograms of the empty vector control. Abbreviations: Var, variant; WT, wild‐type. [file MDC3-8-1140-s006.pdf]
