# Supplementary material for: A Novel Homozygous ADCY5 Variant is Associated with a Neurodevelopmental Disorder and Movement Abnormalities
Source: Mov Disord Clin Pract. 2021 Jul 31;8(7):1140–3. doi: 10.1002/mdc3.13310 (PMC8485619; doi:10.1002/mdc3.13310)
Supplement: Supplementary file 2 — Figure S2. Sanger sequencing electropherograms. First raw—proband (III‐5); second raw—unaffected sister (III‐4); third raw—father (II‐1); fourth raw—mother (II‐2); fifth raw—affected sibling (III‐6); sixth raw—unaffected sibling (III‐1); seventh raw‐unaffected sibling (III‐3). [file MDC3-8-1140-s003.pdf]

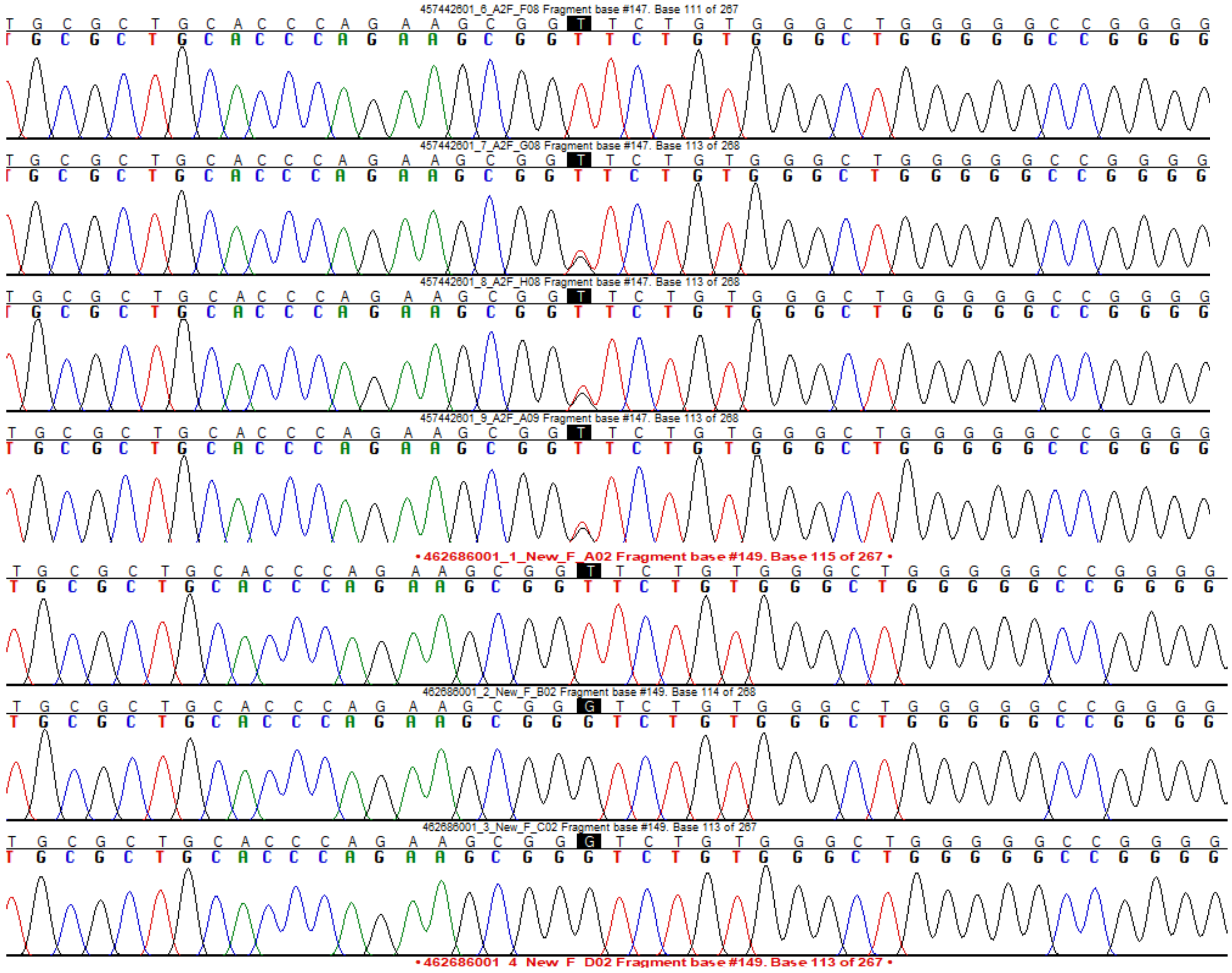

*First raw - proband (III-5); second raw - unaffected sister (III-4); third raw - father (II-1); fourth raw – mother (II-2); fifth raw –affected sibling (III-6); sixth raw – unaffected sibling (III-1); seventh raw-unaffected sibling (III-3)*
