# Supplementary material for: A Novel Homozygous ADCY5 Variant is Associated with a Neurodevelopmental Disorder and Movement Abnormalities
Source: Mov Disord Clin Pract. 2021 Jul 31;8(7):1140–3. doi: 10.1002/mdc3.13310 (PMC8485619; doi:10.1002/mdc3.13310)

A

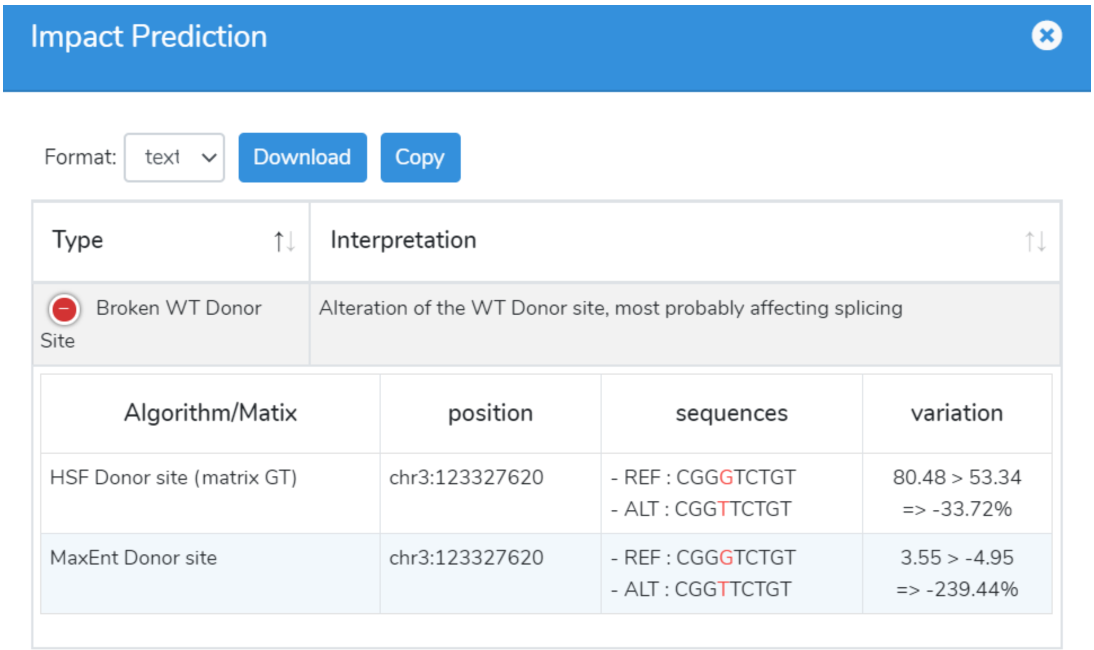
Impact Prediction

Format: text Download Copy

Type	Interpretation
Broken WT Donor Site	Alteration of the WT Donor site, most probably affecting splicing

Algorithm/Matix	position	sequences	variation
HSF Donor site (matrix GT)	chr3:123327620	- REF : CGGGTCTGT<br>- ALT : CGGTTCTGT	80.48 > 53.34<br>=> -33.72%
MaxEnt Donor site	chr3:123327620	- REF : CGGGTCTGT<br>- ALT : CGGTTCTGT	3.55 > -4.95<br>=> -239.44%

B

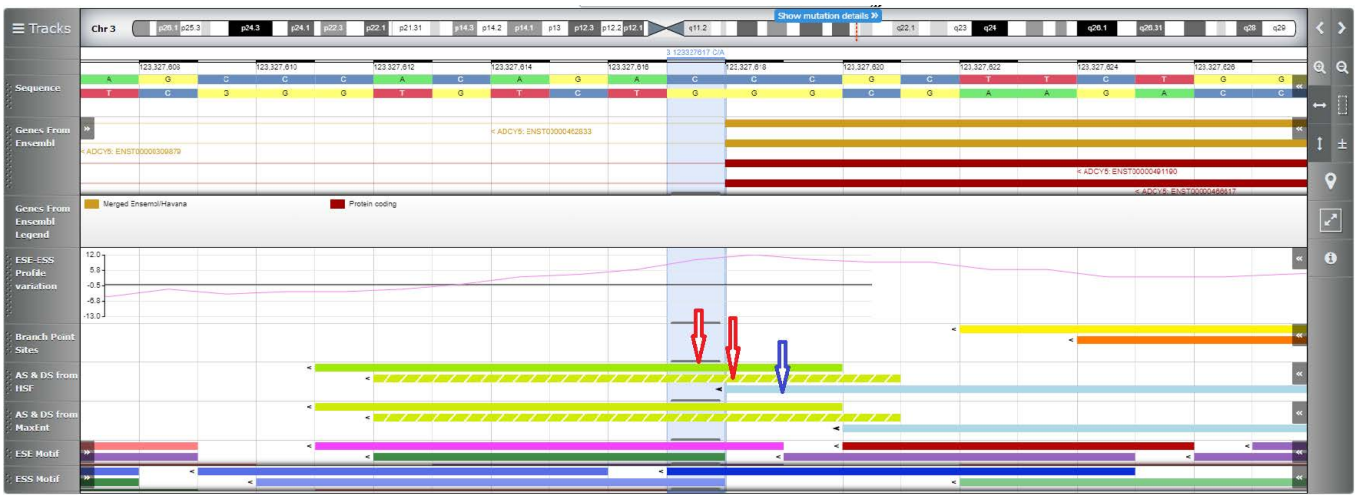

Supplement: Supplementary file 3 — Figure S3. The impact on splicing signals analysis for ADCY5 c.897+1G>T (A, B). Signal interpretation: Broken WT Donor site alteration of the WT Donor site, most probably affecting splicing. The analysis was performed using https://hsf.genomnis.com/. Introns are non‐protein‐coding DNA sequences and those introns immediately adjacent to exons are important for correct splicing between exons. Splicing regions of introns can be divided into Donor and Acceptor sites. The red arrows on Fig. 1B show the Donor site and the blue arrow shows the Acceptor site between the exons 7–8 of the ADCY5 gene. ADCY5 c.897+1G>T variant breaks the Donor site. [file MDC3-8-1140-s007.pdf]
