# Supplementary material for: A Novel Homozygous ADCY5 Variant is Associated with a Neurodevelopmental Disorder and Movement Abnormalities
Source: Mov Disord Clin Pract. 2021 Jul 31;8(7):1140–3. doi: 10.1002/mdc3.13310 (PMC8485619; doi:10.1002/mdc3.13310)
Supplement: Supplementary file 5 — Appendix S1. Minigene assay methods. [file MDC3-8-1140-s008.docx]

**Supplementary Methods**

**Minigene assay**

RNA studies of the *ADCY5* c.897+1G>T variant followed a modified protocol.^1^ Testing ensued by amplifying a 3,927 bp genomic region from control and patient DNA samples that included exons 6-8. Amplification ensued with primers containing *Xho*I and *Bam*HI restriction sites (forward 5’-aattctcgagGGCTGAACGTTCTTGGACTC-3’ and reverse 5’-attggatccCAGACATGCCTGCTTCCAG-3’). Following PCR amplification, and clean-up, restriction enzyme digestion of the PCR fragment and pSPL3 exon trapping vector was performed prior to ligation between exons A and B of the linearized pSPL3-vector. The vector was transformed into DH5α competent cells (NEB 5-alpha, New England Biolabs) that were plated and incubated overnight. The wild-type and variant-containing vector sequences were validated via Sanger sequencing and transfected into HEK293T cells (ATCC) at a density of 2x10^5^ cells per mL. 1 µg of the respective pSPL3 vector was transiently transfected using 3 µl of FuGENE 6 Transfection Reagent (Promega). An empty vector and transfection negative reaction were included as controls. The transfected cells were harvested 24 hours after transfection. Total RNA was prepared using miRNAeasy Mini Kit (Qiagen). Approximately 1 µg of RNA was reverse transcribed using the High Capacity RNA-to-cDNA Kit (Applied Biosystems) following the manufacturer’s protocols. The cDNA was PCR amplified using vector-specific SD6 forward (5’-TCTGAGTCACCTGGACAACC-3’) and SA2 reverse (5’-ATCTCAGTGGTATTTGTGAGC-3’) primers. The amplified fragments were visualized on a 2% agarose gel and Sanger sequenced. cDNA amplicons were cloned and sequence-confirmed following standard protocols for the TA cloning Dual Promoter with pCRII Kit (Invitrogen).

References:

Tompson SW, Young TL. Assaying the Effects of Splice Site Variants by Exon Trapping in a Mammalian Cell Line. Bio Protoc. 2017 20;7(10):e2281.
