# Supplementary material for: A Novel Homozygous ADCY5 Variant is Associated with a Neurodevelopmental Disorder and Movement Abnormalities
Source: Mov Disord Clin Pract. 2021 Jul 31;8(7):1140–3. doi: 10.1002/mdc3.13310 (PMC8485619; doi:10.1002/mdc3.13310)
Supplement: Supplementary file 6 — Table S1. Clinical features of the cases with biallelic ADCY5 variants [file MDC3-8-1140-s001.docx]

|  | **Present report** | | | **6 published cases**  **(Bohlega et al., 2019)** | **2 published cases**  **(Barrett et al., 2017)** | **2 published cases**  **(Okamoto et al., 2021)** |
| --- | --- | --- | --- | --- | --- | --- |
| **Patient** | Patient 1 | Patient 2 | Patient 3 | Data from 6 affected | Data from 2 affected | Data from 2 affected |
| Mutation type | splice donor variant | splice donor variant | splice donor variant | missense | frameshift and missense | missense |
| Mutation at the cDNA level | c.897+1G>T | c.897+1G>T | c.897+1G>T | c.1762G>A | c.409_428del20 (Mat); c.3037C>T(Pat) | c.3712C>T |
| Mutation at the protein level | - | - | - | p.D588N | p.G137Cfs*184 (Mat); p.R1013C (Pat) | p.R1238W |
| Gender | female | female | male | males (3) and females (3) | female and male | female and male |
| Family history | + | + | + | + | + | + |
| Current age | deceased at 4.5 years old | 8 years  old | 2 years  old | 6,8,12,15,18,20 years old | 27 and 24 years old | 3 years old and 1.5 year old |
| **Medical history** | | | | | | |
| Age when involuntary movements were first noted | at 7 months | at 9 months | at 7 months | 2,5,8,10,4,3 years old | 3 years old-in female; 8 years old- in male | NA |
| First symptom (which body part first affected) | limbs | limbs | limbs | oral twitching | limbs | NA |
| Worsening of the involuntary movements over time | + | + | + | + | + | NA |
| Type of progression (rapid, moderate, slow) | slow | slow | slow | slow | slow | NA |
| Initially intermittent movements | + | + | + | 6 (+) | NA | NA |
| Later constant movements | + | + | + | 6 (+) | NA | NA |
| Weight loss | + | + | + | 4 (+), 2 (-) | NA | NA |
| Other | acquired knee arthrogryposis in knees and elbows | acquired knee arthrogryposis in knees and elbows | no | no | no | NA |
| Age of sitting | not achieved | not achieved | not achieved | NA | NA | Delayed 2 (+) |
| Age of walking | not achieved | not achieved | not achieved | delay 4 (+),  2 (-) | 18months -female; 12 month - male | Delayed 2 (+) |

**Table 1.** Clinical features of the cases with biallelic *ADCY5* variants

Table 1 (continued)

| Age of first words/Language abilities | sounds at 2y | only sounds at 1 6/12y | only sounds at 13/12y | delay 3 (+),  3 (+) | delayed, age is not mentioned | Delayed 2 (+) |
| --- | --- | --- | --- | --- | --- | --- |
| **Neurological examination** | | | | | | |
| Cognitive impairment | severely impaired | severely impaired | moderately impaired | 6 (-) | NA | Impaired 2(+) |
| Facial/oral twitching | + | + | + | 6 (+) | NA | NA |
| Axial hypotonia | + | + | + | 6 (+) | NA | 2 (+) |
| Dystonia | + | + | + | 6 (+) | generalized | 2 (+) |
| Distribution of dystonia | extremities and trunk | extremities and trunk | extremities and less trunk | craniocervical, extremities and trunk | extremities and trunk | NA |
| Myoclonus | + | + | + | 6 (+) | yes, superimposed | Stimulus sensitive (1+) |
| Predominant movement disorder | dystonia | dystonia | dystonia | dystonia | dystonia | NA |
| Abnormal gait | couldn't walk | couldn't walk | couldn't walk | 4 (+), 2 (-) | 2 (+) | NA |
| Tremor | + | + | + | 1 (+), 5 (-) | 2 (-) | NA |
| Dysarthria | No speech | No speech | No speech | 6 (+) | NA | NA |
| **Other systems** | | | | | | |
| Cardiomyopathy | + | - | - | only in one affected | NA | NA |
| Episodes of obsessive-compulsive behaviour | + | + | + | 4 (+), 2 (-) | NA | NA |
| Anxiety/ phobias | + | + | + | 5 (+), 1 (-) |  | NA |
| Brain MRI/CT | MRI: Thin corpus callosum mild lateral. | CT: thin corpus callosum, no calcification | MRI: Thin corpus callosum | normal in all | normal in all | mild hypoplasia of the brainstem in male. Normal in female |

NA=not available; CT= computed tomography; MRI=magnetic resonance tomography.
